# An integrative telehealth platform managed by nurses

**DOI:** 10.1186/s13104-022-06197-8

**Published:** 2022-09-17

**Authors:** Vinícius Ynoe de Moraes, César Biselli Ferreira, Camila Kaory Kawagoe, Fernanda Gushken, Guilherme Azevedo, Mário Ferretti Filho

**Affiliations:** Alice Health, Av. Rebouças, 3506 - Pinheiros, São Paulo, SP 05402-600 Brazil

**Keywords:** Telehealth, Mobile health applications, Primary care

## Abstract

**Objective:**

Our aim was to assess the feasibility and preliminary results of implementing a telehealth system, Alice Agora, as a tool for optimizing health delivery in a new primary care-based health system.

**Results:**

We had 4193 consultations over the last 6 months (February and August 2021). Preliminary results show patients high level of satisfaction (Consumer satisfaction score of 4.92). The chief complaints were related to upper respiratory tract (n = 1542; 28.5%), gastrointestinal (n = 781; 14.43%), musculoskeletal (n = 607; 11.22%), and other (n = 643; 11.88%). We found that 20.1% (842) of the cases were solved digitally, that is, by a chat only with a nurse, through the use of health protocols, and 43.9% were solved by nurses with medical assistance. Only 6.6% (277) of the cases had to be referred to the emergency room (ER). This means that 64% of the cases were completely resolved by our nurses-driven system. Forty-eight hours readmission rates were higher for the uncoordinated ER cases compared with the coordinated cases (14.81% vs. 5.87%; p = 0.016). The same pattern was observed for the 72-h readmission rates (16.67 vs. 7.26%; p = 0.02).

## Introduction

Before the COVID-19 pandemic, telehealth in Brazil was limited to interprofessional consultations. It was only during the pandemic that direct patient-doctor consultations were approved, and this meant a unique opportunity to improve accessibility on a large scale [1]. As a consequence, the use of telehealth expanded in public and private clinics and hospitals and included the assistance of health insurance companies. The potential to follow patients remotely started to be better explored, with digital monitoring occurring until clinical improvement; this avoided the burden of health services and unnecessary visits for secondary and tertiary care from the perspective of patient-centered care and integrality of care [2, 3].

Within this context the Alice Health system was created; it is a private health insurance plan based on the principle of integrating a robust primary care health design with a technological structure, allowing clinical decisions to be data-driven. The multilayered design empowers nurses to be at the center of care coordination so that with the support of validated protocols, patients can be continuously guided as they navigate the healthcare system.

Outside Brazil, this model had already been tested, but some challenging aspects such as historical physician-centered care and a lack of control of access to more complex sources of care (such as specialists consultations) were in need to be tested in Latin-American countries [4, 5].

As an important part of the system, Alice Agora was developed as a first-line layer for health care demands. Alice Agora (AA) has an app-based, nurse-driven chat room that addresses any patient complaints related to health issues. In this chat, in which a nurse replies to any queries in a 1–3 min time, evidence-based protocols actions were mapped with the aim of providing quick and primary care-focused resources. From this point, we believe that this made our decisions more prone to deliver care in the most effective way.

Our objectives in this study were to investigate the feasibility and preliminary results of implementing a telehealth system, Alice Agora, as a tool for optimizing health delivery in a new primary care-based health system.

## Main text

### Methods

#### Study design

This study was a prospective cross-sectional study in which the data collection took place between February and August 2021. The data were restricted to the population of a single healthcare insurance plan, Alice, a startup founded in 2020 and based in São Paulo, Brazil. We collected anonymized data from our electronic health record (EHR) and built-in 24/7 chat room.

#### Alice Agora system

Alice Agora is a platform that integrates electronic medical records and a 24/7 chat room, in which patients can communicate with nurses and physicians on duty by message, video, or audio. It is an integrated system based on evidence-based health protocols developed internally that aim to incorporate Alice Agora’s most common chief complaints.

Active and longitudinal communications with patients are the core principles of the platform. A specific communications channel is opened for the associated chief complaint whenever a patient triggers Alice Agora. Nurses and doctors are added to the channel according to the complexity of the case and can decide whether to conduct the case internally or to refer to secondary or tertiary care. Data privacy is preserved and only the patient and the professionals added to the channel have access to the conversation.

#### Data analysis

We based our analysis on patients for whom Alice Agora was available during the period of the study. Due to the descriptive nature of the study, no sample size of observations was calculated. The data collected included the conditions that triggered the activation of the Alice Agora system; the rates of resolution of patient complaints; the rates of satisfaction of patients and health care professionals according to the CSAT (customer satisfaction score) and eNPS (employee net promoter score); and the actions taken after the digital appointment or chat and the resources needed to solve patients’ complaints. For the last component, we stratified the types of resources into seven categories: (1) nursing guidance only, (2) case discussion between nurse and doctor, (3) video consultation with a general physician, (4) referral to the ER, (5) request for a new examination, (6) *asynchronous health support*, or (7) in-person physical examination. *Asynchronous health support* was defined as an interprofessional consultation between a nurse or physician on duty and a medical specialist.

Data analysis was conducted using Excel due to the descriptive nature of the study. Patient data were anonymized for research purposes. Data were presented by descriptive means and percentage rates as appropriate. Consent forms were not applicable due to the populational nature of the analysis. The identified measures were reported as absolute numbers and percentages when relevant. The results of the analysis are presented in tables. We utilized Chi-Square tests to compare data from categorical variables (readmission rates). We considered as significant if alpha was below 5%.

### Results

Our population consisted mostly of females (68.1%), with a median age of 28 (Interquartile range 25–33). Ninety-three percent of our population were considered Healthy, with no concerning condition to be treated: health promotion 67%; self-care support 26%; case management 6%; disease management 1%.

Our results showed a high level of patient satisfaction (average CSAT of 4.92 out of 5.0), with 98% of patients demonstrating confidence about the care received through Alice Agora. A total of 5411 chief complaints were analyzed and yielded 4193 consultations; they are presented in Table 1. A *consultation* was defined as one patient request triggered in the Alice Agora chat, meaning that each case could be associated with more than one chief complaint. For each case, the professionals involved had to closely follow up and monitor the patient remotely.

**Table 1:** Chief complaints that triggered Alice Agora

Category of chief complaint	Total	% of total
Respiratory system	1542	28.50
Digestive system	781	14.43
General and not specific	643	11.88
Musculoskeletal system	607	11.22
Nervous system	522	9.65
Skin	469	8.67
Genitourinary tract	301	5.56
Eyes	186	3.44
Ears	132	2.44
Psychological	94	1.74
Circulatory system	48	0.89
Pregnancy and family planning	41	0.76
Hematology	30	0.55
Endocrine system	12	0.22
Social issues	3	0.06
Total	5411	100

The most prevalent complaints according to the International Classification of Primary Care (ICPC) were related to the respiratory system (n = 138, 28.5%) Table 2 shows hospital admissions by category, with most elective admissions related to surgery and most urgent admissions related to the internal medicine department.

**Table 2:** Hospital admissions by category

Admission category	Type of admission	Total	% of total
Elective	Surgery	58	81.69
	Obstetrics	9	12.68
	Clinical	2	2.82
	Psychiatry	2	2.82
	Subtotal	71	100.00
Urgency	Clinical	34	38.64
	Obstetrics	22	25.00
	Surgery	12	13.64
	Pediatrics	12	13.64
	Neurology	3	3.41
	Orthopedics	3	3.41
	Neonatal	1	1.14
	Urology	1	1.14
	Subtotal	88	100.00

In Table 3 we summarize the distribution of Alice Agora cases in our system. For each care level, we stratified the main pipelines and their chief complaints. We had 3666 cases (87.4%) that were managed by primary care, 250 cases (6.0%) by secondary care, and 277 cases (6.6%) by tertiary care. Nurses were responsible for the screening and follow-up of all cases of acute complaints. Of all the cases, 20.1% were completely managed by on-call nurses with the assistance of protocols. For low-complexity cases that required a medical opinion or the prescription of medications not covered by protocols, the nurse contacted a physician to discuss and manage the case digitally. There were 1840 such cases or 43.9% of all cases. Cases that required a video call with a primary care physician were 17.3% of cases, and those that required asynchronous health support were 4.6% of cases. For non-urgent acute cases that required a physical examination, 6.2% (259 cases) were referred to Alice onsite and 1.4% (58 cases) to a medical specialist’s office. Only 6.6% of cases (277) were referred to the ER. The median service time was 29 min (i.e., from the chat opening until the clinical decision was reached). *Resolution* was defined as clinical stability and improvement, as evaluated by the professionals on duty and by asking the patient if the case was concluded. The median time from the chat opening until resolution was 4 days. It was calculated based on the start time (i.e., the moment the patient sent a message to the Alice Agora app) and case resolution.

**Table 3:** Digital nurses’ participation through primary, secondary, and tertiary care

Care level	% of cases (n)	Pipeline	Chief complaint
Primary n = 3666 (87.4%)	20.1% (842)	Nurse-only guidance by chat, supported by health protocols (nurse online)	Runny nose, common cold
	43.9% (1840)	Nurse and doctor discussion, guidance by chat (physician and nurse online)	Sore throat/bacterial amygdalitis
	17.3% (725)	Nurse triage and video call with physician (nurse and physician online)	Lack of clear communication, need for differential diagnosis
	6.2% (259)	Nurse triage, discussion with physician as needed, and referral to in-person physical examination (Alice onsite)	Cough and fever for 6 days, pneumonia
Secondary n = 250 (6.0%)	4.6% (192)	Nurse triage, discussion with physician as needed, and digital medical report (nurse and specialist online)	Skin and visual complaints
	1.4% (58)	Nurse triage, discussion with physician as needed, and referral to specialist (nurse and specialist office)	Shoulder pain/rotator cuff lesion in MRI
Tertiary n = 277 (6.6%)	6.4% (267)	Nurse triage, discussion with physician as needed, and referral to emergency room (urgent care)	Intense back pain/renal colic
	0.24% (10)	Nurse triage, discussion with physician as needed, and referral to emergency room by ambulance (emergent care)	Thoracic pain, myocardial infarction

ER revisits were calculated to understand whether care coordination exerted an impact on readmission rates. Like any health insurer in Brazil, Alice is not allowed to prevent patients from visiting the ER. The difference is that in the Alice framework patients are encouraged to first consult the Alice Agora team by chat before deciding whether the ER visit is necessary or whether the acute condition can be managed remotely. Of the 412 ER visits analyzed, 358 were coordinated and 54 were uncoordinated. The coordinated visits were those in which patients communicated with health professionals through Alice Agora during and after the case resolution. The uncoordinated visits were those in which patients did not contact Alice Agora before going to the ER, either because of the urgency of the situation or because the patient was not educated about the importance of contacting Alice Agora. We found that the 48-h readmission rates were higher for the uncoordinated ER cases compared with the coordinated cases (14.81% vs 0.5.87%; Chi-Square = 5.74, p = 0.016). The same pattern was observed for the 72-h readmission rates, favoring coordinated cases (16.67 vs. 7.26%; Chi-Square = 5.33; p = 0.02).

### Discussion

The introduction of telehealth services amid the pandemic was promising to increase health access on a national level. The same scenario occurred worldwide and it is more than clear that there are substantial benefits to keeping Telehealth proactively as a part of emergency response [5–7]. There are challenges with respect to maintaining the “routine of telehealth”, as pandemic concerns of social distance have loosened. It is suggested that these actions are in need and are important for the sustainability of Telehealth systems: developing a skilled workforce, empowering consumers, reforming funding; improving the digital ecosystems, and integrating telehealth into routine care [5, 6].

In this sense, the lack of data management systems and the deficit of trained primary care professionals have to be addressed both in public and private providers [7]. This means that health leaders need to ensure proper training and dedicated time slots for administrative tasks or even hire operational teams that can assist those health professionals.

This research piece's hypothesis was that remote 24/7 monitoring and protocols enable the resolution of acute conditions in an effective manner. Our data suggests that 24/7 real-time care coordination may lead to lower ED readmission rates and also decrease unnecessary visits to specialists and excessive laboratory exams. These findings need to be confirmed with a prospective trial.

Along with a clinical support system, a key element in our environment is the empowerment of nurses as coordinators of multidisciplinary teams. With proper autonomy, nurses can be protagonists in care coordination, displaying collaboration, education, counseling, connection, and advocacy [7]. Our shift towards tech-based and protocol-driven care led nurses to decrease their time on “paperwork” and get focused on the care coordination process [8–11].

A hierarchy centered on physicians still prevails in many healthcare organizations and the possibility of collaboration becomes limited. A cultural shift from a physician to a patient-centered environment then becomes essential to ensure a structure moved by continuous improvement of clinical outcomes and operational efficiency.

#### Limitations

This is preliminary data collected in-between our framework was being developed. Our sample consists of a young and disease-free population, which may have made the AA system more feasible. Future prospective studies are in the need to support or refute findings.

## Data Availability

The datasets used and/or analysed during the current study available from the corresponding author on reasonable request.

## Abbreviations

AA: Alice Agora
CSAT: Customer satisfaction score
eNPS: Employee net promoter score
HER: Electronic health record
MRI: Magnetic resonance imaging
ER: Emergency room

## References

[1] Law. https://legis.senado.leg.br/norma/32111272

[2] Caetano R, Silva AB, Guedes ACCM, de Paiva CCN, da Rocha RG, Santos DL (2020). Desafios e oportunidades para telessaúde em tempos da pandemia pela COVID-19: uma reflexão sobre os espaços e iniciativas no contexto brasileiro. Cad Saude Publica.

[3] World Health Organization. https://iris.paho.org/handle/10665.2/28414

[4] Kaundinya T, Agrawal R (2022). Unpacking a telemedical takeover: recommendations for improving the sustainability and usage of telemedicine post-COVID-19. Qual Manag Health Care.

[5] Thomas EE, Haydon HM, Mehrotra A (2022). Building on the momentum: sustaining telehealth beyond COVID-19. J Telemed Telecare.

[6] Smith AC, Thomas E, Snoswell CL (2020). Telehealth for global emergencies: implications for coronavirus disease 2019 (COVID-19). J Telemed Telecare.

[7] Snoswell CL, Chelberg G, De Guzman KR (2021). The clinical effectiveness of telehealth: a systematic review of meta-analyses from 2010 to 2019. J Telemed Telecare.

[8] Lopez AM, Lam K, Thota R (2021). Barriers and facilitators to telemedicine: can you hear me now?. Am Soc Clin Oncol Educ Book.

[9] Doraiswamy S, Abraham A, Mamtani R, Cheema S (2020). Use of telehealth during the COVID-19 pandemic: scoping review. J Med Internet Res.

[10] Grant J, Lines L, Darbyshire P, Parry Y (2017). How do nurse practitioners work in primary health care settings? A scoping review. Int J Nurs Stud.

[11] Labrague LJ, McEnroe-Petitte DM, Leocadio MC, Van Bogaert P, Cummings GG (2018). Stress and ways of coping among nurse managers: an integrative review. J Clin Nurs.

